# Potential of aquatic weeds to improve water quality in natural waterways of the Zambezi catchment

**DOI:** 10.1038/s41598-020-72499-1

**Published:** 2020-09-22

**Authors:** R. Scott Winton, Fritz Kleinschroth, Elisa Calamita, Martina Botter, Cristian R. Teodoru, Imasiku Nyambe, Bernhard Wehrli

**Affiliations:** 1 Institute of Biogeochemistry and Pollutant Dynamics, ETH Zurich, Universitätstrasse 16, 8092 Zurich, Switzerland; 2 Department of Surface Waters, Eawag, Swiss Federal Institution of Aquatic Science and Technology, 6047 Kastanienbaum, Switzerland; 3 Ecosystem Management, Department of Environmental Systems Science, ETH Zurich, 8092 Zurich, Switzerland; 4 Institute of Environmental Engineering, ETH Zurich, 8093 Zurich, Switzerland; 5 Geology Department, School of Mines, University of Zambia, P. O. Box 32 379, Lusaka, Zambia

**Keywords:** Biogeochemistry, Freshwater ecology, Biogeochemistry, Ecology, Environmental sciences

## Abstract

One prominent effect of nutrient pollution of surface waters is the mass invasion of floating plants, which can clog waterways, disrupting human use of aquatic systems. These plants are widely vilified and motivate expensive control campaigns, but their presence may be providing a poorly recognized function in the cycling of excess nutrients. The capacity for floating plants to absorb nutrients from surface water has been understood for decades, primarily from their use in constructed wetlands for wastewater treatment. Yet, in natural settings, there has not been to date any effort to quantify whether floating plant invasions represent important pools or fluxes of nutrients relative to those of the river catchments in which they occur. We found that seasonal hydrologic cycles in the Zambezi trap and flush floating plants from river choke points, such as dams and river confluences, on an annual basis. Peak plant biomass at such choke points constitutes a proxy for estimating annual plant-bound nutrient loads. We assessed the significance of floating vegetation as nutrient sinks by comparing annual plant-bound nutrient loading to conventional river nutrient loading (dissolved and particulate) for four tributaries of the Zambezi River in Zambia. We found that the relative importance of floating vegetation was greatest in the more urbanized catchments, such as the Maramba River draining the city of Livingstone, representing approximately 30% and 9% of annual digestible phosphorus and nitrogen flux respectively. We also found plant-bound phosphorus to be important in the Kafue River (19%), draining the industrial town of Kafue and extensive sugarcane plantations. These results demonstrate the great potential of floating plants to take up excess nutrients from natural river systems. Given the importance of hydrology in the life cycle of floating vegetation, controlled dam discharges may have an important role in managing them and their water quality treatment functions.

## Introduction

Managing water resources in the nexus of energy, food security and environmental health is a major global challenge^1^. This is especially the case in lower income developing countries where urban wastewater treatment infrastructure is limited^2^. One highly visible symptom of nutrient pollution from inadequate wastewater management is the eutrophication of aquatic ecosystems^3^, with far reaching consequences for ecosystem functioning and human well-being. Especially in the warmer regions of the world, a visible symptom of eutrophication is blooms of free-floating aquatic plants ^4^. Such “invasions” are infamous for their conspicuous and costly problems. The plants obstruct boat traffic, clog irrigation schemes, interfere with hydropower operations and threaten fisheries^4^, motivating expensive control campaigns that routinely fail to become sustainable long-term solutions^5–7^.

The control of nutrient pollution sources is widely understood to be an essential component of any strategy to manage invasions of floating plants^8–10^. Some managers have also begun to realize that floating plants are potentially serving an important role in nutrient cycling, removing excess nutrients from surface waters by assimilation into plant tissues^11^. Floating plants, by virtue of their morphology, assimilate nutrients directly from surface waters and have been extensively utilized in constructed wetlands for wastewater treatment^12^. Observations of spiking ambient nutrient concentrations following the shredding or herbiciding of floating plants^13,14^, paving the way for a future bloom, illustrates both the futility of in situ destruction and also the significance of the nutrient pool bound to plant biomass where blooms occur. Floating plants in natural river systems may perform a valuable nutrient uptake function, acting to mitigate the very nutrient pollution that fuels their dominance. Since floating plants can be transported by wind-induced currents, they tend to accumulate at the margins of water bodies where, if water levels drop, they can become stranded on exposed floodplains or lake bottoms. In this way, floating plants can act as a vector transporting nutrients from aquatic to terrestrial settings. However, the significance of floating plants as nutrient sinks relative to local sources and forms has not been explored quantitatively to date.

In order to assess the potential for nutrient uptake by floating plants, we measured seasonal nutrient concentrations in the Zambezi River Basin in Southern Zambia. Surface waters in the study are largely hyperoligotrophic, and yet there have been chronic localized blooms of two invasive floating plants native to South America: Water Hyacinth (*Eichhornia crassipes*) and Amazon Frogbit (*Limnobium laevigatum*). Using a mass balance approach, we estimated the amounts of nutrients contained within floating plant biomass and compared them to conventional estimates of river nutrient loading based on concentration and discharge for four Zambezi tributaries. We used this comparison to estimate the proportion of exported river nutrient loading that is bound to plants and assess whether blooms of floating vegetation could be considered important nutrient sinks in this river system.

In order to understand the landscape and hydrologic context in which floating vegetation invasions occur, we examined remote sensing data on floating vegetation cover and landcover of the catchment area for each tributary to assess whether urban or agricultural sources are most likely drivers of local nutrient pollution and associated floating plant blooms. We also examined time series of rainfall and stream flow to assess the role of hydrology in delivering nutrients from sources to aquatic systems where they can be utilized by floating plants and in transporting the floating plants downstream to system choke points. By linking information from aquatic chemistry, hydrology, and land uses, we provide a systems-thinking approach to the water quality treatment potential of invasive floating vegetation in the rapidly developing Southern African region.

## Methods

### Study area description

**Figure 1:**
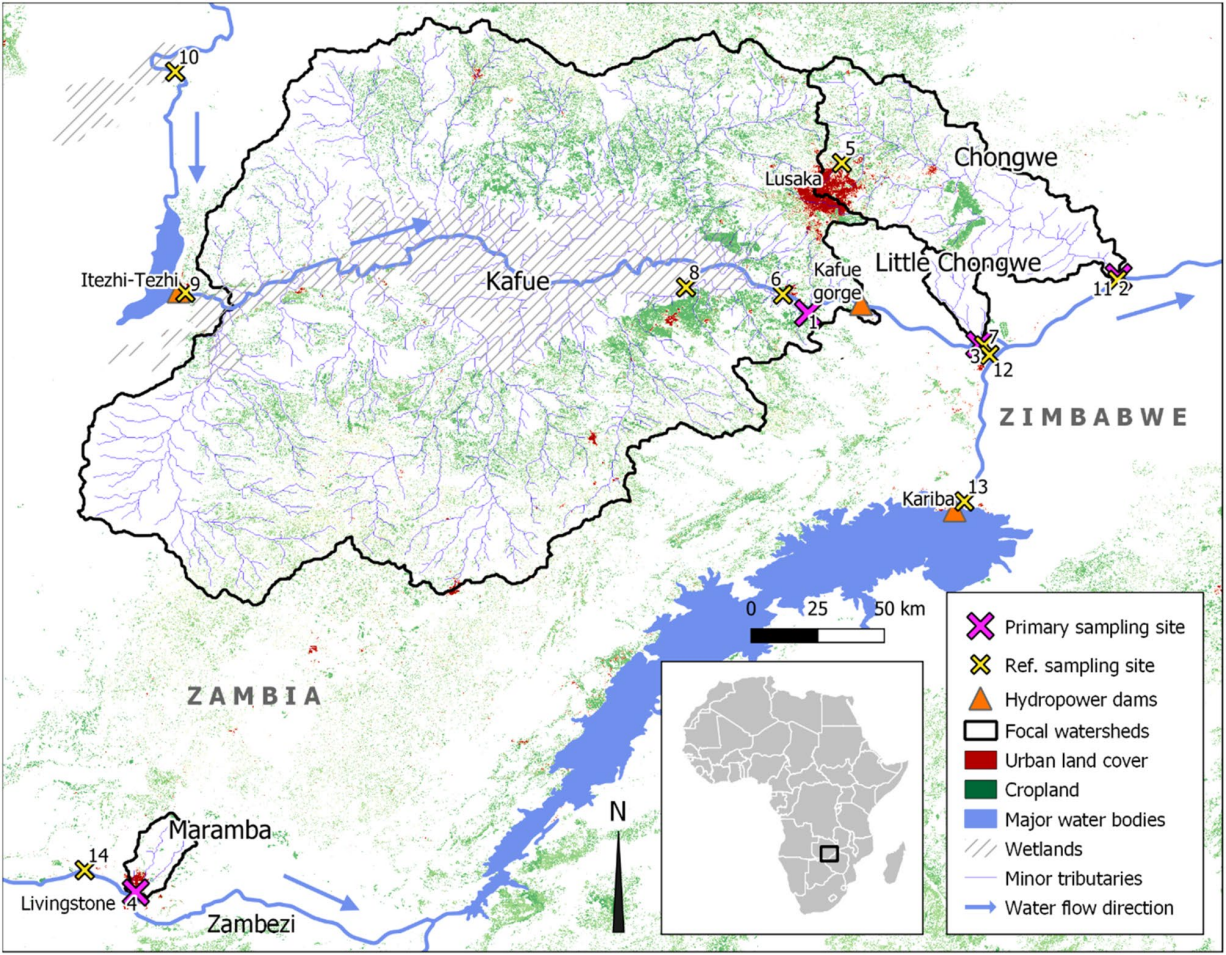
Location of the four catchments: Chongwe, Kafue (subcatchment between Itezhi-Tezhi Dam and Kafue Gorge), Little Chongwe and Maramba, covered in this study with urban and agricultural land cover^15^, wetlands and major dams in southern Zambia (www.openstreetmap.org). The four primary surface water sampling sites (pink) as well as reference sampling sites (yellow) are located through crosses. Map created using QGIS 3.4.11 (https://qgis.org).

The Zambezi is Africa’s fourth largest river and the most important draining into the Indian Ocean. We focused our analysis on four sub-catchments of the Zambezi River in southern Zambia where invasions of Water Hyacinth have been reported in literature and/or which we observed from satellite imagery. These rivers are: Kafue, Chongwe, Maramba and Little Chongwe (Fig. 1). The catchments differ in size and dominant human land-use.

The Kafue River is a major tributary of the Zambezi and is characterized by two major hydropower dams bracketing the large Kafue Flats floodplain: Itezhi-Tezhi Dam upstream and Kafue Gorge Upper Power Station (which we subsequently refer to as simply “Kafue Gorge Dam”) downstream. Operation of these dams has greatly altered the hydrology of the lower reaches of the Kafue River leading to changes in the seasonal biochemical functioning of the Kafue Flats^16,17^ compared to the Barotse Plains, a reference floodplain unmodified by dams^18^. The lower end of the Kafue Flats has expansive coverage of sugar cane cultivation and the downstream river reach above the reservoir formed by the Kafue Gorge Dam (“Kafue Gorge Reservoir”) receives urban and industrial wastewaters from Kafue. Local reports implicate these nutrient sources as drivers of the recurrence of Water Hyacinth blooms in this area including the Kafue Gorge Reservoir. The Kafue River was subject to nutrient loading studies and intensive weed control campaigns from 1998 to 2000^19^. Confusingly, control efforts are described as “ineffective” in the literature^19^, and yet the weed problem abated for a decade from 2001 to 2011. Water Hyacinth is back in recent years, following a seasonal pattern of coverage on the Kafue Gorge Reservoir surface. Mechanical control efforts have also been resumed (personal observations of the authors, February 2019).

The Chongwe River drains one of the more densely populated catchments in Zambia, including parts of the capital, Lusaka, and several nearby townships. After tumbling down the same geographic escarpment as the Kafue, it meets the Zambezi in the Lower Zambezi National Park, approximately 55 km downstream from the confluence of the Kafue with the Zambezi River.

The Maramba River meets the Zambezi just upstream of Victoria Falls and drains a small, but highly urbanized catchment containing most of Livingstone, a popular tourist destination for its convenient access to the falls. According to reports, overflow discharge from Livingstone’s wastewater treatment pools enters the Maramba just upstream of the confluence with the Zambezi^19^. During the latter stages of the dry season, low flows in the Maramba allow the river to build up a dense coverage of Water Hyacinth. These mats are seasonally flushed into the Zambezi when the rains at the end of the dry season restore the flow of the Maramba^20,21^.

The Little Chongwe River meets the Kafue just 8 km upstream of the Kafue’s confluence with the Zambezi. Its small catchment drains a small portion of a large array of pivot irrigation agriculture.

### Assessment of floating vegetation cover

To assess levels of floating vegetation, we used a combination of field surveys and analysis of satellite imagery. Floating vegetation on the Kafue Gorge Reservoir can clearly be detected with Landsat imagery. We used Google Earth Engine to group all available images in the Landsat archive in two-month intervals and extracted floating vegetation cover from 1990 to 2019^22^.

To assess floating vegetation cover on the Maramba, we visually inspected all available historical high-resolution imagery in Google Earth Pro (35 images from 2005 to 2019). We hand-digitized floating vegetation cover over the lower reaches of the river for which channel morphology was clearly and consistently visible and resolvable (2.3 km of linear stream reach). Since there were multiple cases of complete coverage on the Maramba River surface we interpreted complete coverage as a conservative estimate for annual Water Hyacinth biomass export in the catchment.

Relatively few high-resolution satellite images are available in Google Earth Pro for the lower reaches of the Chongwe River and the Little Chongwe (8 each) and they do not provide complete seasonal coverage, making it impossible to apply the same process as for the Maramba. Instead we simply hand-digitized the area of visible floating vegetation from the image with the most cover (16 June, 2016) and added to this an estimate of the area of fringing floating vegetation based on four ground-based inspections of the coverage in 2018 and 2019. We estimated that fringing vegetation has a coverage of 0.5 m wide running for 1.2 km upstream of the confluence with the Zambezi. Hippo presence prevented us from surveying further upstream and we assumed no floating vegetation presence beyond this point, making our estimates conservative.

To convert areal coverage to biomass and nutrient content, we used a synthetic mean of biomass per area from 15 studies and synthetic mean nitrogen and phosphorus content from 14 and 17 studies respectively to estimate the total pool macronutrients bound to Water Hyacinth (Supplemental Tables S1,S2). The nutrient content of Water Hyacinth varies substantially between and within studies, presumably in response to differences in nutrient availability and limitation is experiences as it grows and ages^23^. The Water Hyacinth in Zambian waters spends different parts of its life cycle in very different nutrient settings, ranging from urban wastewater effluent to hyperoligotrophic natural rivers, and we should therefore expect that its nutrient content should also vary substantially in space and time. The synthetic mean nutrient content from varied nutrient settings probably represent a reasonable estimate the nutrient content of Water Hyacinth in Zambia, which grows under a similarly wide range of nutrient conditions. We report uncertainty surrounding mean nutrient content using the standard error of this mean.

### Nutrient sampling

In order to assess the potential for floating vegetation to sequester nutrients from river systems we modelled river nutrient loading for four Zambezi tributaries infested with invasive floating vegetation and estimated the amount of nutrients bound within floating vegetation biomass. We collected surface water samples from these rivers: the Maramba, the Chongwe, the Little Chongwe and the Kafue near the town of Kafue, once every three months for a year starting in March 2018. To evaluate the nutrient environment in the backwaters where we expect Water Hyacinth to be originating, we also sampled two additional sites in the larger catchments in November 2019. In the Kafue Catchment, we sampled two drainage canals conveying industrial wastewater from Kafue. In the Chongwe catchment, we sampled two points on the Gwerere River, an urban stream draining densely populated portions of Lusaka. We sampled additional points on the Kafue and Zambezi Rivers to provide reference nutrient conditions for the region’s major rivers. These sites include the Kafue River: near Hook Bridge, below the Itezhi-Tezhi Dam, near Mazabuka, near Chirundu; and the Zambezi: near Livingstone, below Kariba Dam, near Chirundu, just above Lower Zambezi National Park (for coordinates and exact sampling dates, see Supplemental Table S3).

We passed nitrate and phosphate samples through pre-combusted, pre-weighed glass fiber filters and collected unfiltered samples for analysis of bulk nitrogen and phosphorus content. We collected all samples in triplicate and kept them cool during handling and until analysis in the laboratories at Eawag, Switzerland. We digested unfiltered water samples via autoclave with an alkaline potassium peroxidisulfate solution. We analyzed filtered water samples and digests colorimetrically using a Skalar (Breda, Netherlands) SAN++ automated flow injection analyzer following^24^ and standard procedure ISO 13395:1996. We note that acid digestion of unfiltered surface water is susceptible to underestimation of total phosphorus because of settling of clay particles during sample storage and sub-sampling leading to a bias against potentially phosphorus-rich particulate matter^25^. We assume that such underestimation in our samples is relatively minor because of low stream velocities we observed at our sampling sites, dominance of sandy rather than clay-rich soils in the region, and because we observed low C:N ratios in particulate matter (Supplemental Table S4), indicating a low-density microbial (rather than mineral) composition. Nevertheless, we refer to our P data as “digestible” rather than “total,” to allow for this potential underestimation.

### Discharge calculations, nutrient load estimation and relative importance

We calculated average monthly discharges based on hydrographs collected by the Zambia Electricity Supply Corporation (ZESCO) between 1977 and 2017 for the Kafue Gorge Dam. For the Maramba, Chongwe and Little Chongwe Rivers, we estimated discharge by generating a catchment area: discharge curve using nearby stations from the Global Runoff Data Centre. We estimated rainfall for the Maramba catchment through monthly means from the Climate Hazards Group InfraRed Precipitation with Stations (CHIRPS) dataset^26^.

In order to estimate annual river nutrient loading, we multiplied mean annual discharge by mean digestible phosphorus and mean digestible nitrogen concentrations from our surface water sampling. Our reliance on using the mean of four seasonal concentration values may not fully capture the seasonality of load and is susceptible to effects from outliers. In order to check for evidence of bias from outliers, we also calculated loading based on median concentrations and found that differences translated into a maximum decreased relative importance of plant-bound nutrients of approximately 2%. This is a minor difference compared to other sources of error, such as those stemming from uncertainty in plant biomass per area and nutrient content of plant biomass.

We estimated the relative contributions of plant-bound versus bulk surface water nutrients to riverine nutrient export, by simply calculating the percentage of each relative to their sum. We used the product of standard errors of mean plant biomass and nutrient content values from literature to generate uncertainty envelops around our estimations for the importance of plant-bound nutrients. Here we assumed that the peak plant biomass we detected represents an annual export of intact plant material from each watershed. This assumption provides a conservative underestimate of the importance of plants because the choke points where the plants accumulate seasonally are unlikely to be 100% efficient as traps. Some plant biomass may be exported without being accounted for, but at least in the case of the Maramba and Kafue where hydrologic conditions favor clear trapping and flushing seasons, this is the most reliable approach available to estimate annual plant-bound nutrient export.

### Landcover analysis

The river catchments were extracted from the HydroSheds global database^27^. For the Kafue River, we considered only the catchment below the Itezhi-Tezhi Dam, as we expected the dam to interfere with the nutrient transport from further upstream^28^.

To calculate the area of urban and agricultural land within each catchment we used fractional land cover maps for 2015 from the Copernicus Global Land Service^29^. Using the raster package^30^ in R, we extracted pixel values within each catchment polygon and then calculated the area for all pixels with a fraction of more than 50% for the respective class.

All analyses and figures were completed using R version 3.6.1^31^.

## Results and discussion

### Importance of floating vegetation nutrients

**Figure 2:**
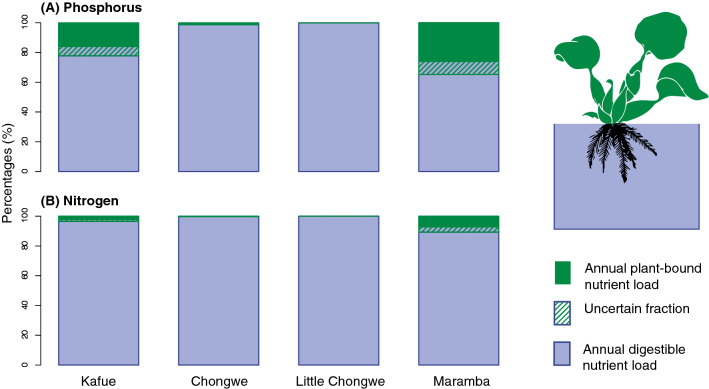
Nutrient loading in four Zambezi tributaries split into plant-bound and digestible river water fractions for Phosphorus and Nitrogen. We conservatively estimate plant-bound nutrient loads from peak biomass detected at choke points (i.e. confluences with major rivers, or in the case of the Kafue, the Kafue Gorge Dam). The uncertain fraction represents compounded standard error of mean biomass per area and mean nutrient content per biomass. Figure created using R 3.6.1^31^ and post-processed with Adobe Illustrator 24.2.1 (https://adobe.com/products/illustrator).

We found that the density of Water Hyacinth and its importance relative to digestible nutrient flux was highly variable among the four study catchments ranging from negligible up to 35% (Fig. 2A,B; Table 1). We found the river with the largest share of annual river P and N flux bound to floating vegetation biomass to be the Maramba. At the other opposite extreme is the Little Chongwe, where we found only trace amounts of floating vegetation during field visits, leading us to conclude that plants are of negligible importance to net nutrient flux in this river. The Kafue and Chongwe Rivers fell between these extremes in terms of its relative importance to nutrient flux.

**Table 1:** Characteristics of the study sub-catchments and maximum amount of floating vegetation detected (Veg. area) and its estimated nutrient content.

River	Q	Catchment	Veg. area	Nutrient content		Plant sink	
	m s^−1^	km^2^	km^2^	P (Mg)	N (Mg)	% P	% N
Kafue	989	44,470	5.5	53.2	263	19.0 [15.9, 22.2]	2.8 [2.3, 3.5]
Chongwe	11	5,130	0.006	0.1	0.3	1.0 [0.8, 1.2]	0.2 [0.2, 0.3]
L. Chongwe	2	530	0	0	0	0	0
Maramba	2	510	0.087	0.8	4.2	30.4 [26.1, 34.7]	8.9 [7.2, 10.7]

Plant sink refers to the amount of plant-bound nutrients as a proportion of measured river nutrient export (confidence intervals in brackets), defined as the sum of annual plant-bound nutrient export and loading based on surface water digestible nutrient concentration and discharge.

We found heavily polluted surface waters in the headwaters of the Chongwe River in Lusaka and in canals draining Kafue industries (Table S1), with P and N concentrations two to three orders of magnitude higher than those of the Zambezi and Kafue (Table 1). These observations demonstrate the presence of urban and industrial nutrient sources capable of driving floating plant blooms in an otherwise nutrient-poor setting (Table 2). The major proportions of nutrients bound to floating plants in the Kafue and Maramba Rivers (Fig. 2; Table 1) relative to summed nutrient flux indicate that these plants are capable of serving as important nutrient sinks. Theoretically, without these plants the amount of non-plant P exported by these rivers would be 19% or 30% greater. This result suggests that invasive floating plants may be helping maintain the oligotrophic character of the large order rivers. This finding is consistent with studies documenting ephemeral increases in surface water nutrients following Water Hyacinth mass mortality elsewhere ^13,14^. In the absence of floating vegetation, other autotrophs (e. g., cyanobacters, algae and phytoplankton) would likely assimilate some portion of the excess nutrients, but a disadvantage of algae and phytoplankton blooms is that their rapid decay drives the hypoxia and fish kills, the classic undesirable impacts of eutrophication brought on by nutrient pollution^32^.

**Table 2:** Nutrient concentrations, discharge and loading for the primary study sites.

River	Map ID	Q_mean_	Concentration		Loading	
		(m^3^ s^−1^)	TP (µg L^−1^)	TN (µg L^−1^)	TP (Mg year^−1^)	TN (Mg year^−1^)
Kafue_town_	1	989	7.3	289	227	9,014
Chongwe	2	11.4	15.6	333	5.6	119
Little Chongwe	3	1.9	14.1	642	0.9	39
Maramba	4	1.9	32.4	718	1.9	43

**Figure 3:**
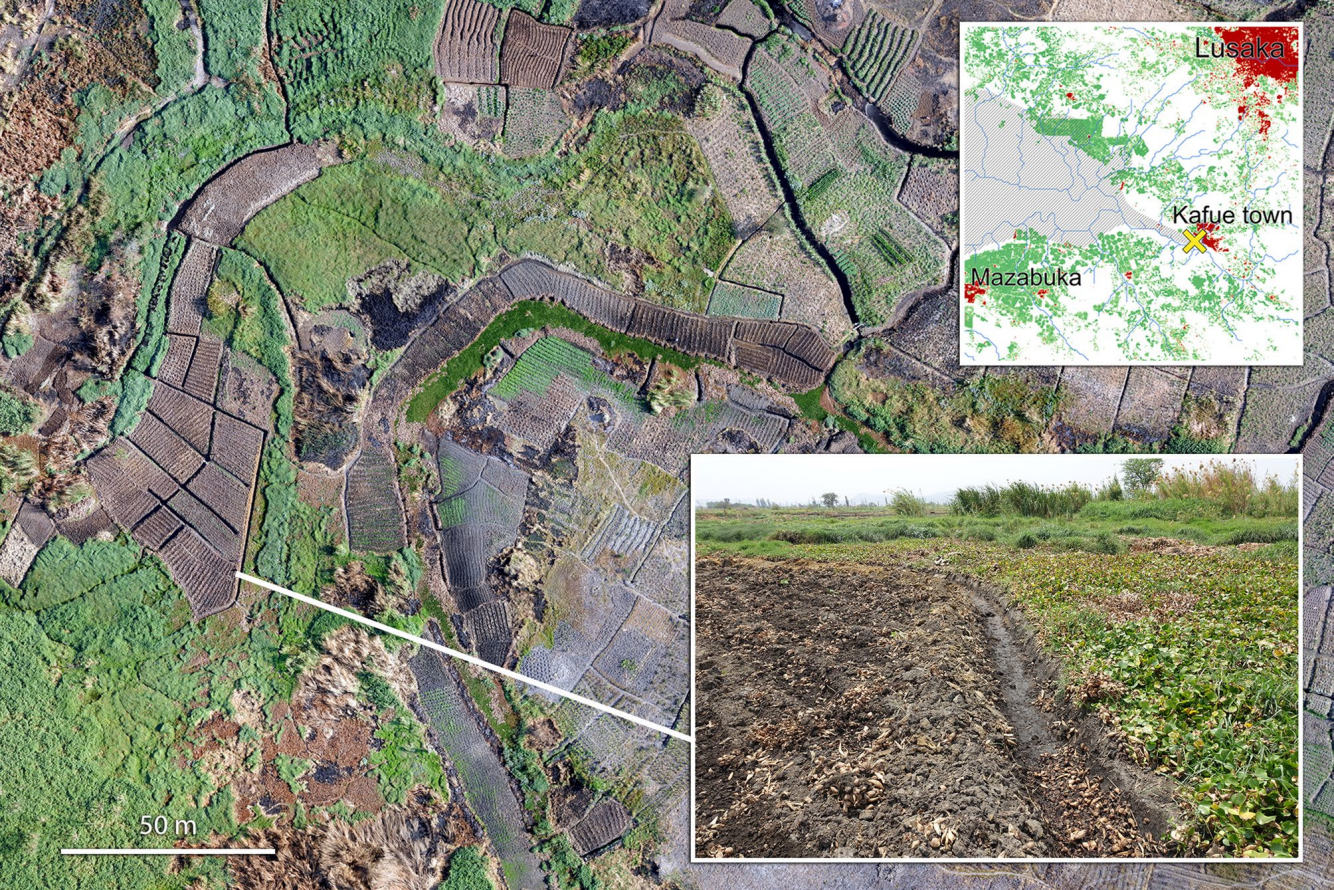
Use of stranded Water Hyacinth for small-scale agriculture near Kafue, Zambia. Large panel: Drone image collected by ATEC 3D; inset small panel: FK. Inset map created using QGIS 3.4.11 (https://qgis.org) with Copernicus data^15^. Image compilation done in Adobe Photoshop 21.2.0 (https://www.adobe.com/products/photoshop).

One advantage of floating plants is that their decomposition is much slower than for algae and phytoplankton. As the plants senesce, they can drift in the direction of flow or be blown by wind, dispersing nutrients away from the source of pollution, even potentially into littoral zones of rivers or lakes where they can act as fertilizer for flood recession agriculture. Water Hyacinth is actively used by farmers as compost along the lower Kafue River (Fig. 3). Vegetation mats removed from the Kafue Gorge Reservoir by hydropower authorities also serves as compost for local farmers (personal observations, November 2019).

The significance of plant-bound nutrient flux may be related to urban cover, with Maramba, draining densely urbanized parts of Livingstone, standing out (Table 3). A comparably-sized catchment without extensive urban cover, the Little Chongwe, supports negligible floating plant biomass despite the presence of some large agricultural pivots that are likely to be a major source of nutrients. Elevated N concentrations for the Little Chongwe are apparent, but not P, suggesting that floating vegetation in the Zambezi Basin may be limited by P availability, as has been proposed in previous studies^33^. Since floating vegetation is present in abundance both upstream and downstream of the Little Chongwe’s confluence with the Kafue there is no reason to suspect that the plants are unable to disperse to this tributary. Compared to the Maramba, the Kafue Catchment has a much lower proportion of urban cover, but floating plants in this larger river still seem to represent a major pool of P. In the Kafue, industrial and/or agricultural sources of nutrients could also be important. The Chongwe Catchment contains 204 km^2^ (4%) of urban area (including some parts of Zambia’s capital, Lusaka) as well as extensive areas under cultivation (11.8%). Yet, the river mouth where our sampling took place does not seem to accumulate floating vegetation as dramatically as in the Maramba or Kafue. This could be because its urban cover lies > 80 km away in the distal headwaters of the catchment and floating plants may not survive the tumultuous journey off the edge of the Central Zambian Plateau as the Chongwe drops 600 m before meeting the Middle Zambezi where we made our observations. It is also possible that the plants simply do not accumulate at the confluence between the Chongwe and the Zambezi, leading us to underestimate the amount of floating plant biomass and the significance of its nutrients within this sub-catchment. Because the amplitude of seasonal changes in water level is heavily dampened and controlled by Kariba Dam, the Zambezi downstream does not cause as strong seasonal backwater effects in side channels as it does in its unregulated upper reaches above Victoria Falls.

**Table 3:** Land cover within the four Zambezi study sub-catchments, southern Zambia.

River	Catchment area (km^2^)	Urban area (km^2^)	Cropland area (km^2^)	Urban ratio (%)	Cropland ratio (%)	Distance to urban (km)
Chongwe	5128	204.2	603.1	4.0	11.8	82
Maramba	507	31.4	14.5	6.2	2.9	2
Little Chongwe	528	0.2	6.5	0.03	1.2	-
Kafue	44,466	464.31	6257.1	1.0	14.1	16

### Invasions over time

**Figure 4:**
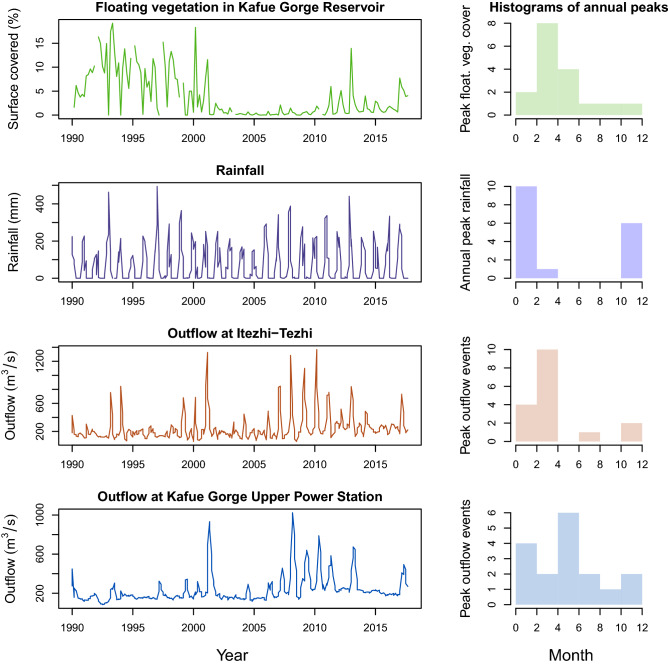
Time series of floating vegetation coverage in the Kafue Gorge Reservoir and of local hydrology (ZESCO data). Histograms of annual peaks highlight how the dominant seasonality of floating plant occurrence is governed by rainfall and discharge seasonality. Typical peak floating vegetation cover (March to June) is preceded by local rainfall peaks (October to January) that flush nutrients into backwaters stimulating floating plant growth. Peak flows in the Kafue upstream of the Kafue Gorge (February to April, as indicated by Itezhi-Tezhi outflows) drives floating vegetation out of backwaters and into the Kafue Gorge. Plant cover in the reservoir declines during peak Kafue Gorge flows (April to June), which flush plants through the spillway. Figure done in R 3.6.1^31^.

**Figure 5:**
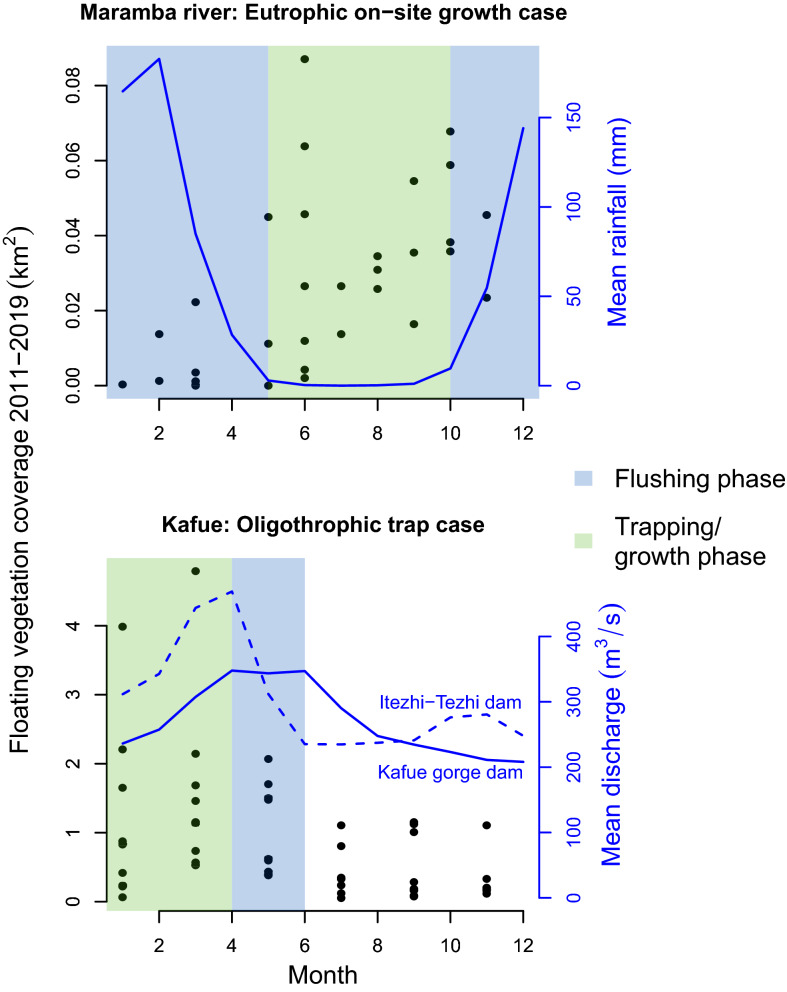
Contrasting seasonal patterns of floating vegetation cover in relation to hydrologic and nutrient context of the Maramba and Kafue Catchments. In the dry season, the Maramba River does not flow and floating vegetation is trapped by backwater effects at the confluence with the Zambezi, allowing plants to progressively accumulate (and grow under favorable nutrient conditions) until local rainfall flushes them out at the start of the wet season. In the Kafue Gorge Reservoir plants accumulate as the Kafue water level rises, flushes vegetation out of nutrient rich backwaters. Plants are flushed through the spillway, mechanically removed and/or senesce and sink from April to June during peak outflows at the Kafue Gorge Power Station. Each floating vegetation cover data point represents a remote sensing observation (Landsat for Kafue Gorge; Google Earth Pro for Maramba River) made between 2011 and 2019. Mean monthly discharge of Kafue Gorge and Itezhi-Tezhi (ZESCO data) are spanning 2011 through 2017. Mean rainfall is for Maramba catchment from CHIRPS global rainfall data 2011–2019^26^. Figure done in R 3.6.1^31^.

The floating vegetation of the Kafue, because it is effectively trapped by a hydropower dam forming the Kafue Gorge Reservoir, can be readily detected and counted from space. Analysis of satellite imagery from 1990 to present reveals three distinct periods of vegetation prevalence (Fig. 4): 1990 to 2001 had high coverage with seasonal oscillations in extent, but some floating vegetation persisting year round; 2002 to 2010 had minimal coverage with some minor seasonal peaks; 2012 to 2018 had highly seasonal coverage with peak coverage comparable to the 1990s, but no year round persistence. The period of high coverage in the 1990s is corroborated by contemporaneous reporting^19^. The sparse cover during the 2000s appears to be a result of integrated nutrient control and plant removal campaigns following a national emergency declaration in 1998^11^. The cause for the floating plants reemergence after 2012 is not clear, but throughout the time series there is evidence of seasonality, which tends to coincide with peak flows in the Kafue (Fig. 4). Local rainfall, which typically arrives a month before peak flow is also likely important for flushing nutrients into backwaters creating conditions favorable for Water Hyacinth growth. The rising Kafue flood waters flush out these backwaters, dislodging floating plant mats that can be exported downstream and accumulate in the Kafue Gorge Reservoir (Fig. 5).

An examination of the Water Hyacinth cover at the Maramba River reveals a different seasonal modality. Here, the floating vegetation coverage is greatest during the end of the dry season (Fig. 5). There are two reasons for this pattern: a) urban effluents from the Livingstone urban area are least diluted by rainwater during the dry season leading to the best conditions for plant growth, and b) low flows and backwater effects from the Zambezi do not allow the floating plants to escape into the Zambezi .

These seasonal patterns of Water Hyacinth cover demonstrate the importance of hydrology in governing the timing and severity of invasions. Floating vegetation is accumulating and moving through the river systems in a predictable manner based on river flow and possibly local rainfall. This finding may have important implications for management strategies since the river flow in both the Kafue and Zambezi is regulated by hydropower operations. Currently there is a severe problem at the Kafue Gorge Reservoir with mats of plants and associated organic sediments clogging intakes and fouling turbines (personal observations, November 2019). The rivers in this study, especially the Kafue, provide essential services for southern Zambia, including hydropower, 50% of the municipal water supply for Lusaka, irrigation water, and fisheries. As a result, several Zambian governmental and private agencies collectively manage the rivers evaluated in this study. We recommend that these stakeholders, as part of their own self-directed integrated water resource management efforts, consider new strategies regarding floating vegetation. Specifically, the regulation of flows (i.e. from hydropower infrastructure) has the potential to flush floating vegetation out of areas where it is problematic and concentrate it in areas where its water treatment function is desirable or where it is at least less of a nuisance. This approach would fit into the environmental flows framework, which has been integrated into Itezhi-Tezhi Dam operations strategy since its construction^34^. The interaction between environmental flows and floating vegetation dynamics has not been explored to date, but maintaining high water levels in the Kafue Flats during favorable wind conditions could allow vegetation to drift into floodplains and become stranded. This would not only help fertilize the floodplains, but also help reduce the pools of plants and nutrients in primary channels, effectively mitigating the most notorious effects they cause, such as blocked waterways and clogged irrigation and hydropower infrastructure. The possibility of harvesting the plants from concentration points for production of biofuels and/or compost could also be a strategy for reducing the plant burden on the downstream Kafue Gorge Dam and at the same time mitigate the problem of biowaste^35^. Hydrologic management as a means to control floating plant invasions would represent a new approach to an old problem and merits further investigation.

## Supplementary information

Supplementary Information
