# Supplementary material for: Potential of aquatic weeds to improve water quality in natural waterways of the Zambezi catchment
Source: Sci Rep. 2020 Sep 22;10:15467. doi: 10.1038/s41598-020-72499-1 (PMC7508817; doi:10.1038/s41598-020-72499-1)
Supplement: Supplementary file 1 — Supplementary Information [file 41598_2020_72499_MOESM1_ESM.docx]

**Supplementary information for: Potential of aquatic weeds to improve water quality in natural waterways of the Zambezi catchment**

R. Scott Winton*, Fritz Kleinschroth*, Elisa Calamita, Martina Botter, Cristian R. Teodoru, Imasiku Nyambe, Bernhard Wehrli

*equal contributions

## Analysis of Kafue Canal water samples

In addition to the spectrophotometric methods described in the main text, we also analyzed samples for ions and cations using a Metrohm (Herisau, Switzerland) 882 Ion Chromatograph Compact Plus with an 889 IC Sampler System. We report NH_4_-N results from the ion chromatograph for the Kafue Canals because high concentrations made digestion for total nitrogen unreliable (Supplemental Table 3).

## Analysis of particulate matter

We oven dried and weighed glass fiber filters to calculate total suspended solids. Subsequently we fumigated them in a desiccator with HCl vapor to remove the inorganic carbon fraction and then analyzed them for carbon and nitrogen content on a ThermoFinnigan FlashEA 1112 coupled to a DeltaV Advantage Continuous-Flow IRMS.

**Supplemental Table 1.** Areal Water Hyacinth (*Eichornia crassipes*) biomass (dry).

| **Biomass (kg m^-2^)** | **Reference** |
| --- | --- |
| 2.13 | (Boyd and Scarsbrook 1975) |
| 2.07 | (Gopal et al. 1978)^*^ |
| 3.46 | (Gopal 1987) |
| 2.40 | (Knipling et al. 1970)^*^ |
| 2.11 | (Lallana 1980)^*^ |
| 2.21 | (Lallana 1981) |
| 3.20 | (Neiff et al. 1977)^*^ |
| 1.50 | (Penfound and Earle 1948) |
| 1.28 | (Penfound 1956) |
| 1.40 | (Perez del Viso et al. 1968)^*^ |
| 2.31 | (Reddy and Tucker 1983) |
| 1.73 | (Sabattini et al. 1983)^*^ |
| 0.72 | (Sahai and Sinha 1970) |
| 0.63 | (Singh and Sahai 1979)^*^ |
| 2.97 | (Wooten and Dodd 1976)^*^ |
| **2.01** | **mean** |
| 0.83 | standard deviation |
| 0.21 | standard error |
| 10.63 | residual standard error (%) |

^*^Values as reported in Gopal (1987). We were unable to access the original texts.

**Supplemental Table 2.** Water Hyacinth (*Eichornia crassipes*) nutrient content.

| **N (%)** | **P (%)** | **Reference** | |
| --- | --- | --- | --- |
| 1.03 | 0.43 | (Abdalla and Abdel Hafeez 1969) | |
| - | 0.53 | (Abdelhamid and Gabr 1991) | |
| 1.43 | 0.59 | (Abou el Fadl et al. 1970)^*^ | |
| - | 1.10 | (Agrupia 1953)^*^ | |
| 2.50 | 0.42 | (Boyd 1969) | |
| 2.64 | 0.43 | (Boyd 1970)^*^ | |
| 2.39 | 0.54 | (Boyd and Vickers 1971) | |
| 2.20 | 0.44 | (Oliveira 1977)^*^ | |
| - | 0.50 | (Easley and Shirley 1974) | |
| 1.18 | 0.26 | (Gunnarsson and Mattsson 1997) | |
| 1.83 | 0.17 | (Howard-Williams and Junk 1976) | |
| 3.12 | 0.43 | (Musil and Breen 1977) | |
| 1.61 | 0.31 | (Parra and Hortenstine 1974) | |
| 2.76 | 0.53 | (Poddar et al. 1991) | |
| 2.90 | 0.50 | (Polprasert et al. 1980)^*^ | |
| 2.90 | 0.63 | (Wolverton and Mcdonald 1978) | |
| 5.01 | 0.38 | (Xie et al. 2004) | |
| **2.38** | **0.48** | **mean** |  |
| 1.00 | 0.20 | standard deviation |  |
| 0.27 | 0.05 | standard error |  |
| 11.28 | 9.94 | residual standard error (%) |  |

^*^Values as reported in Gopal (1987). We were unable to access the original texts.

**Supplemental Table 3.** Nutrient concentration data, sampling dates and coordinates for all study sites.

| River | Map ID | Sampling date | Concentration | | Latitude | | Longitude |
| --- | --- | --- | --- | --- | --- | --- | --- |
|  |  | *yyyy_mm_dd* | *TP (µg L^-1^)* | *TN (µg L^-1^)* | *Decimal degrees* | | |
| Kafue_town_ | 1 | 2018_03_18 | 13 | 304 | 15.837 °S | 28.237 °E | |
|  |  | 2018_07_06 | 7.4 | 299 |  |  | |
|  |  | 2018_11_12 | 7.9 | 253 |  |  | |
|  |  | 2019_02_12 | 1.2 | 29 |  |  | |
| Chongwe | 2 | 2018_03_21 | 18.7 | 400 | 15.709 °S | 29.339 °E | |
|  |  | 2018_06_27 | 10.1 | 237 |  |  | |
|  |  | 2018_10_26 | 8.2 | 252 |  |  | |
|  |  | 2019_02_14 | 2.6 | 50 |  |  | |
| Little Chongwe | 3 | 2018_03_22 | 24.3 | 1127 | 15.948 °S | 28.844 °E | |
|  |  | 2018_06_27 | 8.2 | 352 |  |  | |
|  |  | 2015_10_26 | 8.8 | 236 |  |  | |
|  |  | 2019_02_14 | 2.2 | 342 |  |  | |
| Maramba | 4 | 2018_03_16 | 22.3 | 660 | 17.889 °S | 25.854 °E | |
|  |  | 2018_11_01 | 25.7 | 212 |  |  | |
|  |  | 2019_02_21 | 13.2 | 855 |  |  | |
| Gwerere_1^*^ | 5 | 2019_11_07 | 6,580 | 730 | 15.331 °S | 28.331 °E | |
| Gwerere_2^*^ | 5 | 2019_11_07 | 2,250 | 15,800 | 15.304 °S | 28.358 °E | |
| Kafue canal_1^†^ | 6 | 2019_11_25 | 510 | 76,000 | 15.771 °S | 28.149 °E | |
| Kafue canal_2^†^ | 6 | 2019_11_25 | 12,300 | 6,680 | 15.771 °S | 28.149 °E | |
| Kafue_Chirundu_ | 7 | 2018_03_20 | 22.0 | 327 | 15.951 °S | 28.862 °E | |
|  |  | 2018_06_26 | 10.0 | 228 |  |  | |
|  |  | 2018_10_27 | 8.9 | 228 |  |  | |
|  |  | 2019_02_13 | 1.2 | 52 |  |  | |
| Kafue_Mazabuka_ | 8 | 2018_03_17 | 9.3 | 381 | 15.745 °S | 27.805 °E | |
|  |  | 2018_07_07 | 6.2 | 325 |  |  | |
|  |  | 2018_11_11 | 7.9 | 248 |  |  | |
|  |  | 2019_02_19 | 2.4 | 27.7 |  |  | |
| Kafue_Itezhi-Tezhi_ | 9 | 2018_03_24 | 12.7 | 441 | 15.764 °S | 26.030 °E | |
|  |  | 2018_06_05 | 4.9 | 249 |  |  | |
|  |  | 2018_11_09 | 6.6 | 196 |  |  | |
|  |  | 2019_02_26 | 4.1 | 37.3 |  |  | |
| Kafue_Hook_Bridge_ | 10 | 2018_03_25 | 8.7 | 247.7 | 14.981 °S | 25.994 °E | |
|  |  | 2018_06_30 | 4.4 | 194 |  |  | |
|  |  | 2018_11_07 | 9.4 | 95.0 |  |  | |
|  |  | 2019_02_25 | 1.8 | 37.3 |  |  | |
| Zambezi_Lower_ | 11 | 2018_03_21 | 14 | 399 | -15.719 °S | 29.335 °E | |
|  |  | 2018_06_27 | 7.7 | 229 |  |  | |
|  |  | 2018_06_28 | 7.4 | 259 |  |  | |
|  |  | 2018_10_26 | 7.1 | 234 |  |  | |
|  |  | 2019_02_14 | 3.3 | 53.7 |  |  | |
| Zambezi_Chirundu_ | 12 | 2018_03_22 | 7.7 | 233 | 15.985 °S | 28.881 °E | |
|  |  | 2018_06_26 | 6.6 | 191 |  |  | |
|  |  | 2018_10_25 | 5.1 | 250 |  |  | |
|  |  | 2019_02_13 | 2.7 | 58.0 |  |  | |
| Zambezi_Kariba_ | 13 | 2018_03_18 | 3.7 | 311 | 16.504 °S | 28.792°E | |
|  |  | 2018_06_29 | 5.5 | 196 |  |  | |
|  |  | 2018_10_28 | 6.3 | 223 |  |  | |
|  |  | 2019_02_17 | 1.5 | 44.7 |  |  | |
| Zambezi_Livingstone_ | 14 | 2018_03_16 | 5.7 | 206 | 17.810 °S | 25.673 °E | |
|  |  | 2018_07_03 | 4.1 | 186 |  |  | |
|  |  | 2018_11_01 | 8.1 | 220 |  |  | |
|  |  | 2019_02_22 | 3.7 | 34.3 |  |  | |

^*^At the Gwerere we only measured dissolved nutrients, so these values actually refer to PO_4_-P and NO_3_-N rather than the respective totals.
^†^Nitrogen concentrations were too high for accurate quantification via spectrophotometric methods in the Kafue canals. The TN values instead refer to NH_4_-N measured via ion chromatograph.

**Supplemental Table 4.** Mean total suspended solids and particulate carbon and nitrogen content of Zambian study sites from four campaigns in 2018 and 2019.

| River | TSS mg/L | %C_particulate_ | %N_particulate_ | C:N |
| --- | --- | --- | --- | --- |
| Maramba | 27.7 | 7.7 | 1.3 | 5.8 |
| Chongwe | 10.1 | 9.1 | 1.4 | 6.4 |
| Little Chongwe | 12.7 | 6.6 | 1.1 | 5.8 |
| Kafue | 3.3 | 16.1 | 2.4 | 6.7 |

**References**

1. Boyd, C. E. & Scarsbrook, E. Influence of nutrient additions and initial density of plants on production of waterhyacinth Eichhornia crassipes. *Aquat. Bot.* **1**, 253–261 (1975).

2. Gopal, B., Sharma, K. & Trivedy, R. Studies on ecology and production in Indian freshwater ecosystems at primary producer level with emphasis on macrophytes. in *Glimpses of Ecology* (eds. Singh, J. & Gopal, B.) 349–376 (International Science Publications, 1978).

3. Gopal, B. *Water Hyacinth*. (Elsevier B.V, 1987).

4. Knipling, E., West, S. & Haller, W. Growth characteristics, yield potential, and nutritive content of water hyacinths. *Soil Crop Sci Soc Fla Proc* **30**, 51–63 (1970).

5. Lallana, V. H. Productividad de Eichhornia crassipes (Mart.) Solms. en una Laguna Isleña de la Cuenca del Río Paraná Medio. II. Biomasa y dinámica de población. *Ecología* **5**, 1–16 (1980).

6. Lallana, V. H. Productividad de eichhornia crassipes. *Bol. la Soc. argentina Bot.* **20**, 99–107 (1981).

7. Neiff, A. P. de, Neiff, J. & Bonetto, A. Enemigos naturales de Eichhornia crassipes en el nordeste argentino y posibilidades de su aplicación al control biológico. *Ecosur* **4**, 137–156 (1977).

8. Penfound, W. T. & Earle, T. T. The biology of the water hyacinth. *Ecol. Monogr.* **18**, 447–472 (1948).

9. Penfound, W. T. Primary Production of Vascular Aquatic Plants. *Limnol. Oceanogr.* **1**, 92–101 (1956).

10. Perez del Viso, R., Tur, N. M. & Mantovani, V. Estimación de la biomasa de hidrófitos en cuencas isleñas del Paraná. *Physis* **28**, 219–226 (1968).

11. Reddy, K. & Tucker, J. Productivity and nutrient uptake of water hyacinth, Eichhornia crassipes I. Effect of nitrogen source. *Econ. Bot.* **37**, 237–247 (1983).

12. Sabattini, R. A., Lallana, V. H. & Marta, M. C. Inventario y biomasa de plantas acuáticas en un tramo del valle aluvial del río Paraná Medio. *Rev. la Asoc. Ciencias Nat. del Litoral* **14**, 179–191 (1983).

13. Sahai, R. & Sinha, A. B. Contribution to the ecology of indian aquatics. *Hydrobiologia* **35**, 376–382 (1970).

14. Singh, S. & Sahai, R. Seasonal changes in the biomass of Eichhornia crassipes (Mart) solms in’Jalwania’pond of Gorakhpur [India]. *Indian J. Ecol.* **6**, 30–34 (1979).

15. Wooten, J. W. & Dodd, J. D. Growth of water hyacinths in treated sewage effluent. *Econ. Bot.* **30**, 29–37 (1976).

16. Abdalla, A. A. & Abdel Hafeez, A. T. Some aspects of utilization of water hyacinth ( *Eichhornia crassipes* ). *PANS Pest Artic. News Summ.* **15**, 204–207 (1969).

17. Abdelhamid, A. M. & Gabr, A. A. Evaluation of water hyacinth as a feed for ruminants. *Arch. für Tierernaehrung* **41**, 745–756 (1991).

18. Abou el Fadl, M. *et al.* Utilization of water-hyacinth as an organic manure with special reference to water borne helminths. *J. Microbiol. United Arab Repub.* **3**, 27–34 (1970).

19. Agrupia, F. Value of water hyacinth as silage. *Philipp. Agric. J.* **37**, 50–56 (1953).

20. Boyd, C. E. The nutritive value of three species of water weeds. *Econ. Bot.* **23**, 123–127 (1969).

21. Boyd, C. E. Chemical analyses of some aquatic vascular plants. *Arch. fur Hydrobiol.* **67**, 78–85 (1970).

22. Boyd, C. E. & Vickers, D. H. Variation in the elemental content of Eichhornia crassipes. *Hydrobiologia* **38**, 409–414 (1971).

23. Oliveira, S. Produtividade primaria e evapotranspiracao da baronesa (Eichhornia crassipes) e alface d’agua (Pistia stratiotes) em condicoes de clima tropical [Bahia. (Univ. Federal da Bahia, 1977).

24. Easley, J. F. & Shirley, R. L. Nutients elements for livestock aquatic plants. *Hyacinth Control J.* **12**, 82–85 (1974).

25. Gunnarsson, C. & Mattsson, C. *Water hyacinth – trying to turn an environmental problem into an agricultural resource*. *Minor Field Studies-Swedish University of Agricultural Sciences, International Office (Sweden)* (1997).

26. Howard-Williams, C. & Junk, W. J. The decomposition of aquatic macrophytes in the floating meadows of a central Amazonian várzea lake. *Biogeographica* **7**, 115–123 (1976).

27. Musil, C. F. & Breen, C. M. The influence of site and position in the plant community on the nutrient. *Hydrobiologia* **53**, 67–72 (1977).

28. Parra, J. V. & Hortenstine, C. C. Plant Nutritional Content of Some Florida Waterhyacints And Response By Pearl Millet to Incorporation of Waterhyacinths in three soil types. *Hyacinth Control J* **12**, 85–90 (1974).

29. Poddar, K., Mandal, L. & Banerjee, G. C. Studies on water hyacinth (Eichhornia crassipes) - chemical composition of the plant and water from different habitats. *Indian Vet. J.* **68**, 833–837 (1991).

30. Polprasert, C., Wangsuphachart, S. & Muttamara, S. Composting nightsoil and water hyacinth in the tropics. *Compost Sci. Util.* **21**, 25–27 (1980).

31. Wolverton, B. C. & Mcdonald, R. C. Nutritional composition of water hyacinths grown on domestic sewage. *Econ. Bot.* **32**, 363–370 (1978).

32. Xie, Y., Qin, H. & Yu, D. Nutrient limitation to the decomposition of water hyacinth (Eichhornia crassipes). *Hydrobiologia* **529**, 105–112 (2004).
